# Multimodal, label-free imaging to elucidate radiation resistance mechanisms

**DOI:** 10.1117/1.BIOS.1.3.030502

**Published:** 2024-12-27

**Authors:** Irene Georgakoudi

**Affiliations:** Dartmouth College, Thayer School of Engineering, Hanover, New Hampshire, United States

## Abstract

The commentary remarks on recent work that demonstrates the power of multimodal, label-free imaging to elucidate radiation resistance mechanisms and provides a useful method for plotting multivariable imaging data, uncovering metabolic heterogeneity bulk analyses would miss.

The resistance of head and neck tumors to radiation therapy remains a significant challenge in cancer treatment, with mechanisms still not fully understood.^1^ Metabolic adaptation, regulated by hypoxia-inducible factor 1-alpha (HIF-1α), plays a likely role in conferring this resistance.^2^ Hypoxia often stabilizes HIF-1α, driving glycolysis and other survival pathways. However, tumor metabolism exhibits substantial spatial heterogeneity, and traditional bulk measurements obscure critical localized differences. This limitation underscores the need for methodologies that can resolve metabolic function at high spatial resolution.

In their study, Ivers et al.^3^ address this issue by employing label-free two-photon excited fluorescence imaging to assess the optical redox ratio (ORR), defined here as $\frac{I_{FAD}}{I_{NAD(P)H}+I_{FAD}}$ alongside fluorescence lifetime imaging microscopy (FLIM) of NAD(P)H, followed by co-registered immunohistochemical assessments of hypoxia (through pimo labeling) and HIF-1α levels. These studies are performed on frozen tissue sections acquired from tumors established from either radiation resistant (*UM-SCC-47)* or radiation sensitive (*UM-SCC-22B)* cells. Such sections are acquired prior to treatment and at 24 and 48 hours following treatment (with no treatment controls included during all time points). Bulk assessments from the entire tissue sections, reveal limited differences and mechanistic insights. This is only achieved with more nuanced considerations of the heterogeneity of metabolic function based on the detailed pixel-wise distributions of the ORR values along with hypoxia and HIF1α levels. An innovative way of displaying such multi-variable information is presented by the authors, as indicated in Fig. 1(left panel). In this manner, distinct tumor regions are identified within which elevated HIF-1α levels are associated with either low or high ORR values. The latter group is indicative of non-hypoxic HIF-1α stabilization, potentially driven by reactive oxygen species (ROS). Notably, the authors find that in radiation resistant tumors there is a higher prevalence of regions with low ORR values and high bound NAD(P)H intensity fractions (Fig. 1, right panel), consistent with enhanced fatty acid synthesis, which has been shown to promote radiation resistance in previous studies.^4^^,^^5^

**Fig 1:**
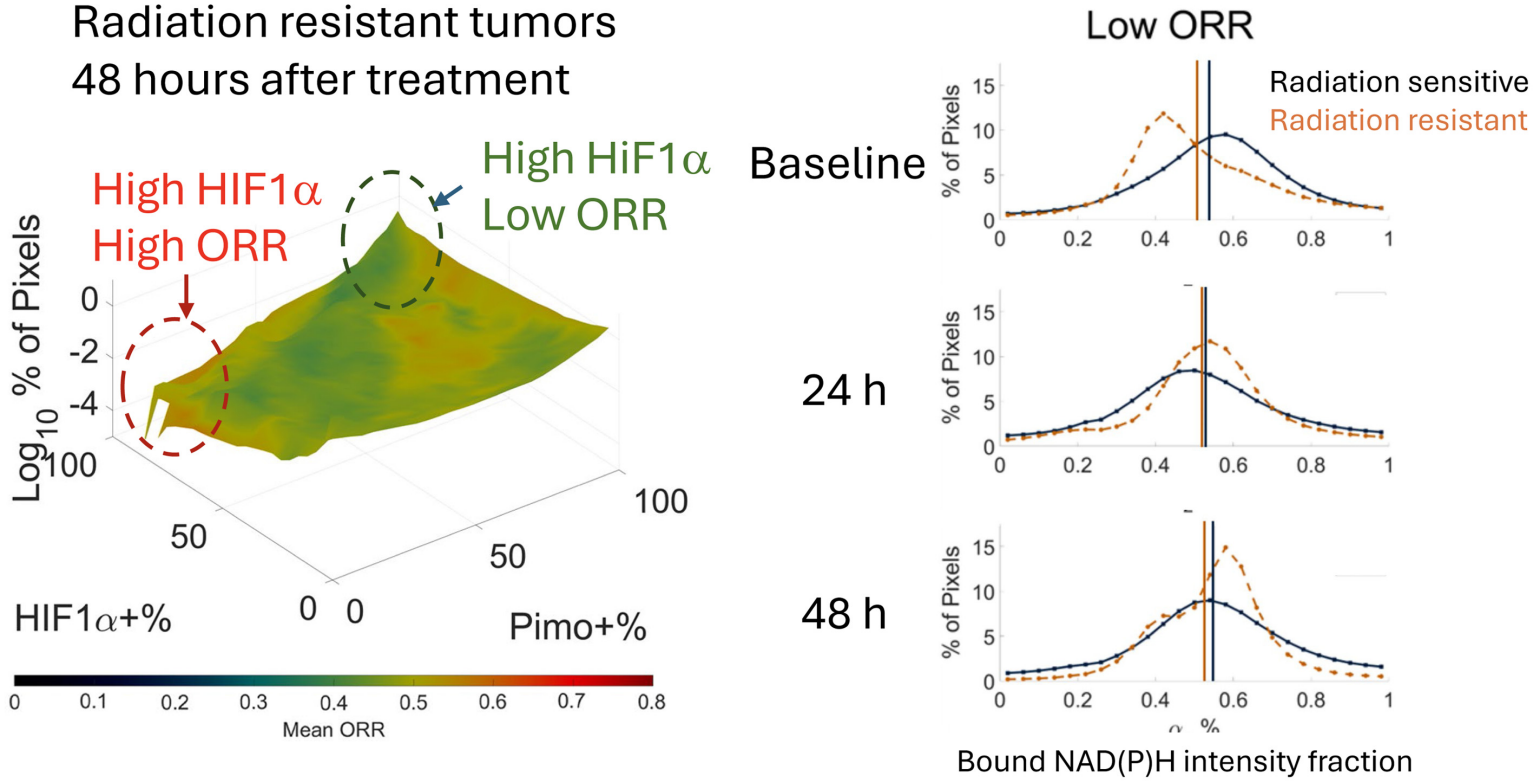
Left panel: Representative multi-variate histogram (from Fig. 6 in Ref. 3) representing simultaneously changes in HIF1α, hypoxia, and ORR and highlighting the high levels of metabolic heterogeneity in these tumors. Right panel: Changes in the Bound NAD(P)H intensity fraction distributions are significant in regions of low ORR in radiation resistant tumors. Such changes are detected only when the detailed distribution shapes are considered.

Despite its contributions, the study has limitations. Quantitative comparisons from a larger number of specimens, which could highlight the prevalence of localized metabolic function differences between treatment and control groups and between radiation sensitive and resistant tumors, would be useful for making more clear connections between the nature of the detected changes and radiation resistance. The influence of other fluorophores, such as lipofuscin, on ORR and FLIM measurements warrants further scrutiny. Finally, the reliance on frozen-thawed tissue sections potentially introduces artifacts, even though the authors have taken reasonable measures to limit them.

Nonetheless, the authors demonstrate the power of multi-modal, label-free imaging to elucidate radiation resistance mechanisms and provide a useful method for plotting multi-variable imaging data. Their approach uncovers metabolic heterogeneity that bulk analyses would miss, emphasizing the importance of spatial and multivariate contexts. Future work could build on this by enabling *in vivo*, dynamic measurements over time, providing a more comprehensive view of tumor response to therapy. This study exemplifies the potential of advanced imaging modalities to reveal critical insights into cancer biology. By bridging spatial resolution with functional metabolic assessments, it opens pathways for better understanding and overcoming treatment resistance in clinical settings.
